# Arthroscopic anatomical double-bundle anterior cruciate ligament reconstruction for asian patient using a bone-patellar tendon-bone and gracilis tendon composite autograft: a technical note

**DOI:** 10.1186/1758-2555-4-9

**Published:** 2012-03-14

**Authors:** Takuya Tajima, Etsuo Chosa, Keitaro Yamamoto, Nami Yamaguchi

**Affiliations:** 1 Division of Orthopedic Surgery, Department of Medicine of Sensory and Motor Organs, Faculty of Medicine, University of Miyazaki, 5200 Kihara, Kiyotake, Miyazaki 889-1692, Japan

**Keywords:** Anterior cruciate ligament, Double bundle reconstruction, Resident's ridge, Bone patellar tendon bone graft, Gracilis tendon graft

## Abstract

**Background:**

Recent years have seen anterior cruciate ligament (ACL) reconstruction being performed in a broad range of patients, regardless of age, sex and occupation, thanks to great advances in surgical techniques, surgical instruments and basic research. In cases of ACL reconstruction, bone-patellar tendon-bone (BTB) graft or hamstring graft are frequency used. However, potential complications associated with tunnel enlargement due to soft tissue graft such as hamstring were reported. On the other hand, an altered rotational axis resulting in significantly greater translation of the lateral compartment in the single bundle compared with double bundle ACL reconstruction was reported.

**Method and procedure:**

A reconstruction procedure was modified for the ACL using a double bundle that is the combination of BTB and gracilis tendon composite autograft. Two tibial and two femoral bone tunnels are used to reconstruct two bundles of ACL; an anteromedial bundle (AMB) and a posterolateral bundle (PLB). The femoral bone tunnels are created just posterior to the resident's ridge. The tibial bone tunnels are created at the center of AM and PL tibial attachment, respectively. BTB is fixed in the AM tunnels produced on the anatomical points of tibia and femur. The gracilis graft is fixed in an anatomical PL tunnel produced. The mean width of BTB is 7 mm, since10 mm graft is sometimes not suitable for patients, especially small Asian people and females. For these patients, 10 mm graft is bigger than one third of patella tendon width.

**Conclusion:**

The devised surgical procedure based on a combination of BTB and gracilis autograft is suitable reconstruction method for patients who have small or medium width of patellar tendon such as Asian people and females. This technique is also applicable to revision surgery.

## Introduction

Anterior cruciate ligament (ACL) injury is a common injury in sports activity. In most cases, ACL laxity causes knee joint instability in sports activities such as cutting or pivoting, which can lead to articular cartilage degradation and/or meniscus injury. ACL reconstruction is also often needed to prevent secondary osteoarthritis [1-3].

Recent years have seen ACL reconstruction being performed in a broad range of patients, regardless of age, sex and occupation, thanks to great advances in surgical techniques, surgical instruments and basic research [4]. In cases of anterior cruciate ligament reconstruction, bone-patellar tendon-bone (BTB) graft or hamstring graft are frequency used [5-9]. However, potential complications associated with tunnel enlargement due to soft tissue graft such as hamstring were reported [10,11]. In cases of ACL reconstruction with BTB graft, the bone tunnels are filled with bone plug in order to decrease the risk of tunnel enlargement. On the other hand, an altered rotational axis resulting in significantly greater translation of the lateral compartment in the single bundle compared with double bundle ACL reconstruction was reported [12]. To meet these necessities, the reconstruction method was modified using a double bundle technique with BTB and gracilis autograft. BTB corresponds to the anteromedial bundle of the ACL, and gracilis tendon corresponds to the posterolateral bundle of the ACL; this technique presents an alternative method for anatomical double bundle ACL reconstruction. Written informed consent was obtained from the patient for publication of this report and any accompanying images.

### Surgical procedures

#### Arthroscopic evaluation

Surgery is performed under general anesthesia with the patient in supine position on the operating table. The arthroscopy is inserted into the joint cavity through the anterolateral portal which is adjacent to external edge of the patellar tendon. The ACL remnant, meniscus and articular cartilage conditions are carefully confirmed.

#### Graft harvesting and preparation

**Figure 1:**
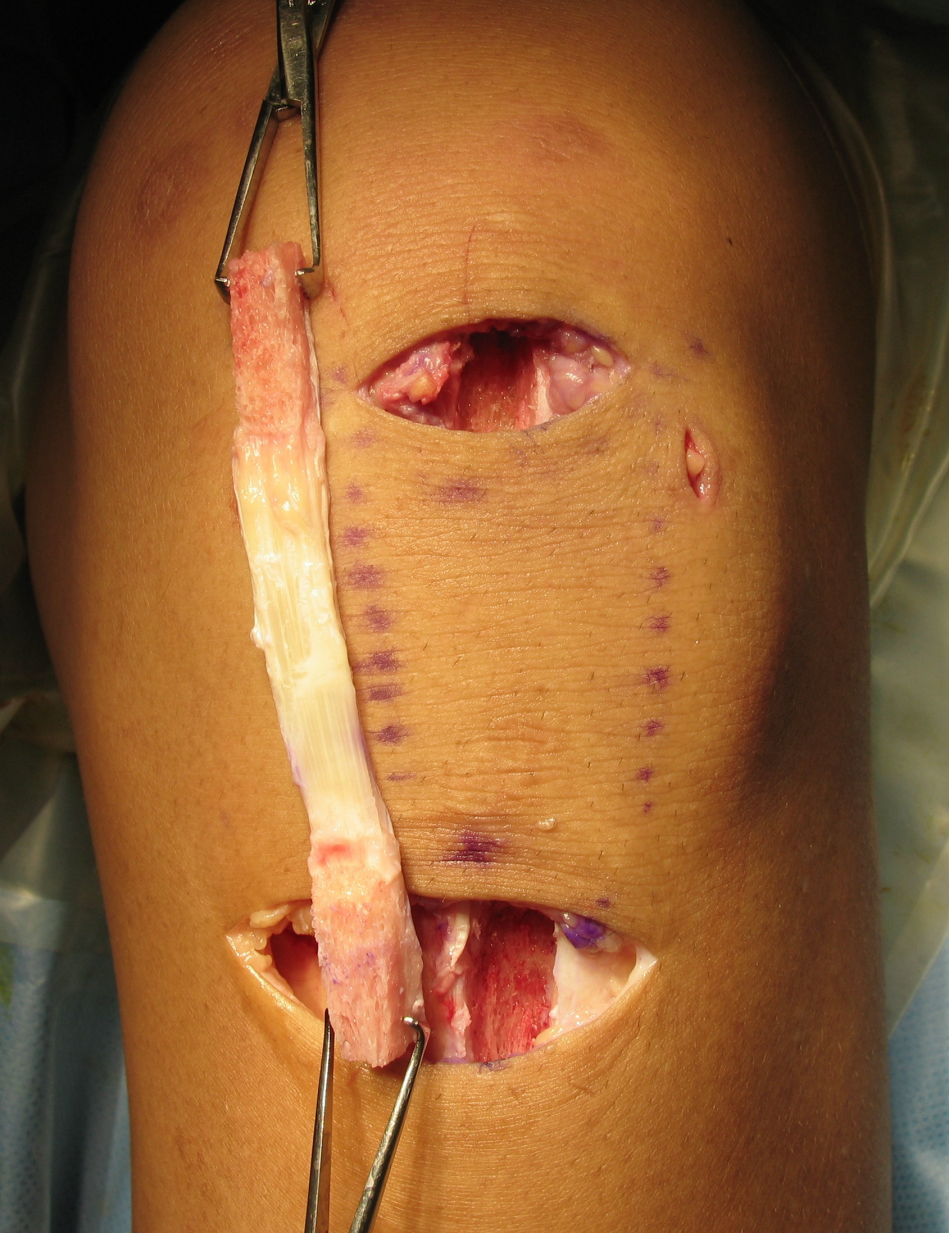
**Skin incision**. Two transverse skin incisions and harvested bone patellar tendon bone (BTB) graft. A 3 cm long proximal incision is placed on the distal side of the patella, and a 4.5 cm long distal incision on the tibial tuberosity.

Two transverse skin incisions are made in the ipsilateral knee. A 3 cm long proximal incision is placed on the distal side of the patella and a 4.5 cm long distal incision on the tibial tuberosity (Figure 1). A central third of the patellar tendon with attached patellar and tibial bone plug autograft is harvested with subcutaneous tunneling technique with the aim of leaving the infrapatellar nerve undamaged and the major part of the paratenon nonincised according to Kartus's method [13], and is harvested with following dimensions; a rectangular shaped bone plug on patella of width 7 mm, length 20 mm, and depth 7 mm. The defect in the tendon is left open. Both sides of the tissue sample are trimmed and prepared to allow it to pass easily within the 7 mm diameter. EndoButton-CL-BTB (Smith & Nephew, Andover, MA) is attached on the femoral side through the 1.8 mm diameter hole which is created on the bone plug, and 2 FiberWires (Arthrex, Naples, FL) are attached on the tibial side.

Gracilis tendon graft harvesting is performed through the same distal incision which is described above. Sartorius fascia is incised along the course of the gracilis. Gracilis tendon is mobilized using blunt finger dissection. Once the tendon is free from adhesion, gracilis tendon is harvested using a tendon stripper. The double or triple folded gracilis tendon is carefully prepared to allow it to pass within the 5 or 5.5 mm diameter. Endobutton-CL (Smith & Nephew, Andover, MA) is attached on the femoral side. The baseball grove suture with a fiberwire is performed on the tibial side

#### Notch preparation

The anteromedial portal and an additional accessory far anteromedial portal are made. The soft tissues in the notch are roughly excised with punch or mechanical instruments. The remaining fibrous tissues on the lateral notch wall are delicately removed using radiofrequency device. Care was taken to completely preserve undulation of the bony surface around the attachment area. After clearage of the lateral notch wall, a meticulous effort is made to find out a linear ridge in posterior one-third of the lateral notch wall. The bony ridge called resident's ridge is a useful landmark for anatomical femoral tunnel drilling in arthroscopic ACL reconstruction [14]. Usually, notch plasty is not performed.

#### Tunnel preparation

**Figure 2:**
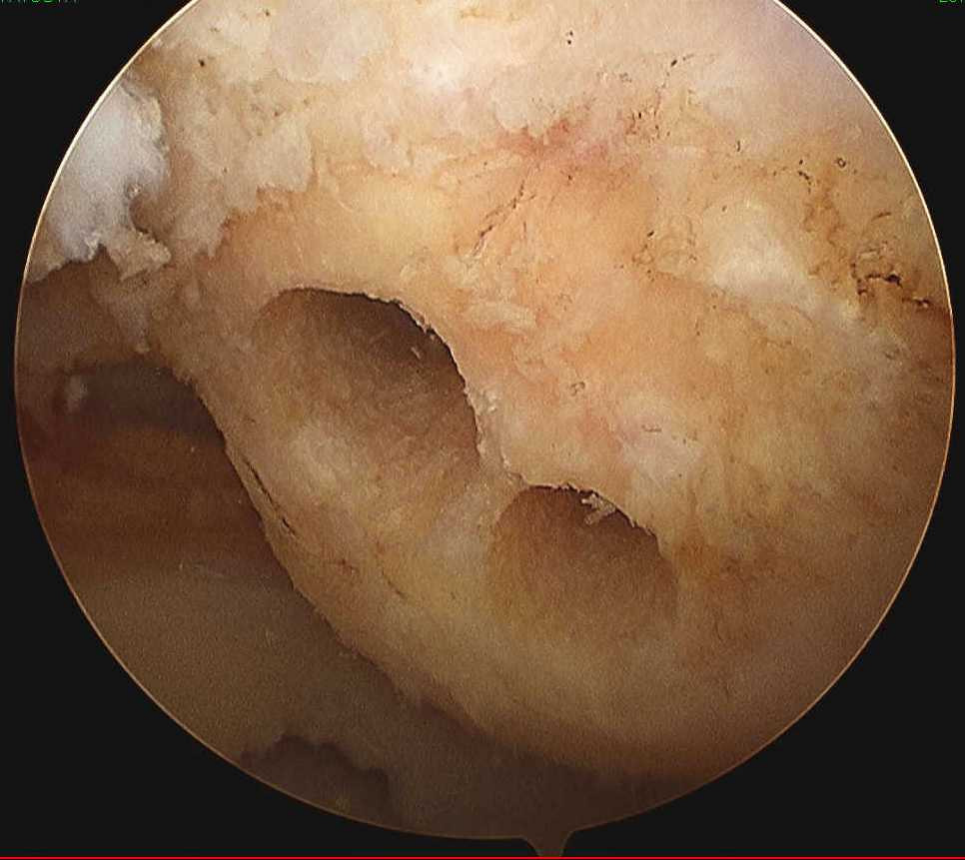
**Femoral tunnels**. Arthroscopic findings of position of the femoral tunnels. Two independent bone tunnels are created just behind the resident's ridge.

For femoral tunnel preparation, a guide pin is inserted using the ACL guide system into the joint from the far-medial portal. Two independent sockets and tunnels are created just posterior to the resident's ridge in inside-out fashion with the knee flexed beyond 135 degree. After tunnels are drilled using a 4.5 mm cannulated reamer along the guide pin, a tunnel of anteromedial bundle of width 7 mm, and a tunnel of posteolateral bundle of width 5-5.5 mm are created using a transportal technique (Figure 2). A tunnel of anteromedial bundle is positioned at the 1:30-o'clock orientation for the left knee or at 10:30-o'clock for the right knee [4]. A tunnel of posterolateral bundle is positioned at the 2:30-0'clock orientation for the left knee or at 9:30-o'clock for the right knee.

**Figure 3:**
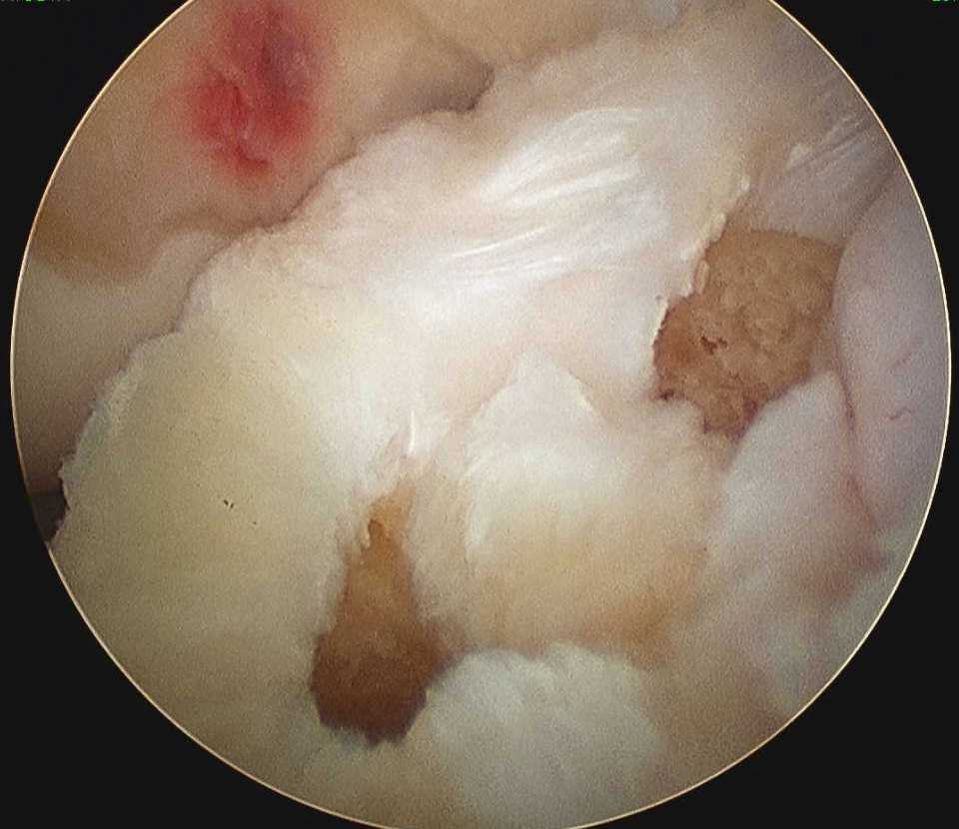
**Tibial tunnels**. Arthroscopic findings of position of the tibial tunnels. Two independent bone tunnels are created in the attachment of anterior cruciate ligament (ACL) The length of the tunnel is measured with scaled probe, and suitable size of Endobutton-CL and CL-BTB are determined, respectively.

To insert a guidewire for tibial tunnel preparation, a tibial aimer (Smith & Nephew, Andover, MA) was used. The aimer tip portion is introduced into the joint cavity through the medial infrapatellar portal with the knee flexed 90 degree. The tibial aimer tip portion is placed at the center of the posteolateral bundle attachment on the tibia, which is located at the most posterior aspect of the area between the tibial eminences, and 5 mm anterior to the posterior cruciate ligament at the anatomical positions proposed by Yasuda *et al. *[4]. A guide wire 2 mm in diameter is drilled in the tibia. Next, a guide wire for the anteromedial bundle is inserted with the same aimer. The tibial aimer tip portion is placed at the center of the tibial attachment of the anteromedial bundle. Careful observation is made to confirm that the guide wires are placed at right position and do not penetrate the medial collateral ligament. Two independent bone tunnels are created; a tunnel of anteromedial bundle of width 7 mm, and a tunnel of posteolateral bundle of width 5-5.5 mm (Figure 3).

#### Graft passage and fixation

**Figure 4:**
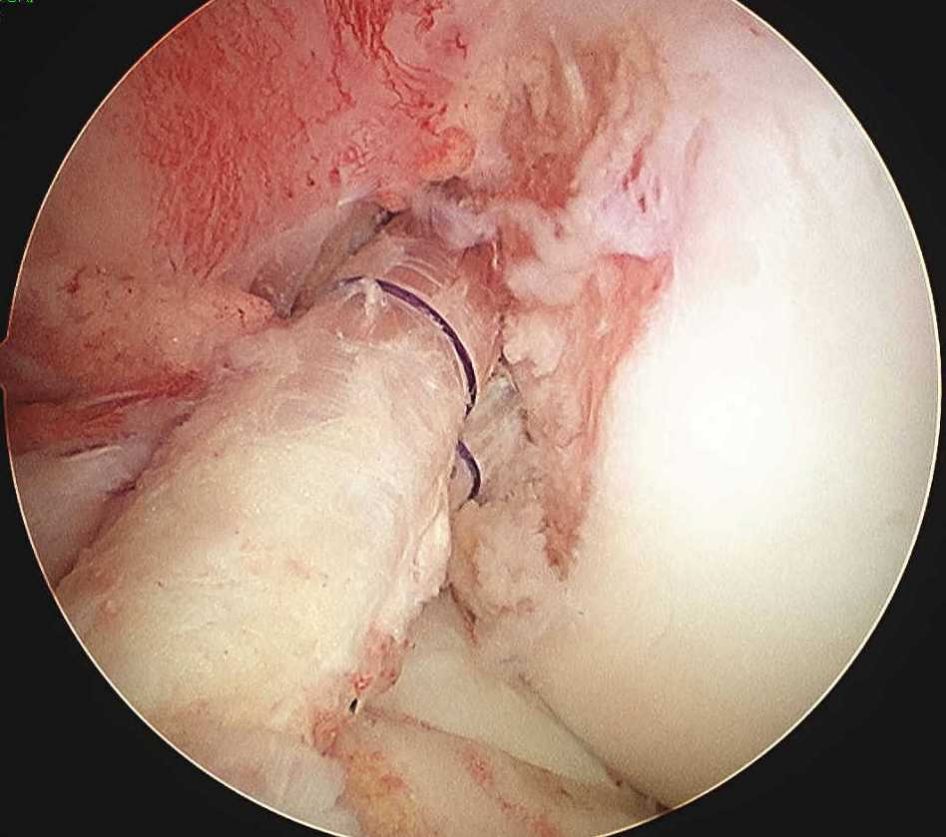
**Arthroscopic findings of reconstructed ACL double bundle**.

**Figure 5:**
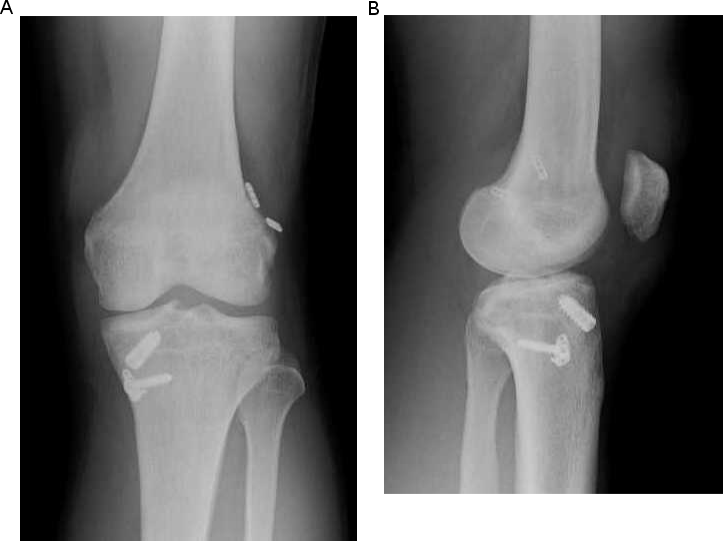
**A and B Postoperative radiograph**. A: anterior to posterior view, B: lateral view.

The gracilis tendon graft for the posterolateral bundle is introduced through the tibial tunnel to the femoral tunnel using a passing pin. The Endobutton is flipped on the femoral cortical surface. Next, the BTB graft for the anteromedial bundle is placed in the same manner. Thus, the 2 bundles are intra-articularly placed with defferent directions (Figure 4). The bone plug in the anteromedial tibial tunnel is fixed with 6-7 mm Softsilk screw (Smith & Nephew, Andover, MA) with the knee in 20 degree of flexion applying maximum manual traction. An assistant surgeon simultaneously applies a tension of 40 N to the gracilis tendon graft for posteolateral bundle in the tibial tunnel using the tensiometer, and the graft is fixed to tibia using double spike plate system (Meira, Aichi,Japan) with the knee in a position of 20 degree flexion (Figure 5A and 5B).

## Discussion

Recently, ACL reconstructive procedures such as endoscopic anatomical double bundle technique using hamstring graft which are passed through two tibial and two femoral bone tunnels have improved and reported excellent outcome [4]. Moreover, in Bedi's report, an altered rotational axis resulted in significantly greater translation of the lateral compartment in the anatomical center-center single bundle ACL reconstruction compared with anatomical double bundle ACL reconstruction [12]. On the other hand, patients with ACL deficient knees that were treated with hamstring graft showed tunnel widening [10,11]. BTB is one of the popular grafts used for ACL reconstruction. The advantages of using the BTB graft include strength, ready availability, strong bone to bone fixation, and prompt healing of the bone plugs. However, sometimes, over 10 mm graft is not suitable for patients, especially small Asian people and females. For these patients, 10 mm graft is bigger than one third of patella tendon width [15].

The present procedure is anatomical graft route double bundle reconstruction using 7 mm BTB graft for anteromedial bundle. BTB with a bone block at the both ends of graft is applied to minimize tunnel enlargement. The disadvantage and limitation of present procedure is not only harvesting the gracilis tendon, but also the BTB, which thus increases the risk of harvest site morbidity. However, the Sartorius and semitendinosus tendons will function well and that graft site morbidity can be minimized if the gracilis tendon alone is harvested. In terms of the tension load for soft tissue graft in double bundle ACL reconstruction, manual maximum does not recommended [16]. However, the tension load for BTB graft in double bundle technique is still controversial. In the present procedure, BTB graft is applied with maximum manual traction according to Hara's method [17]. After an average 15 months follow-up of sixteen Asian patients (thirteen males and three females) treated using this technique, the preliminary post-operative clinical results showed no definite laxity. The bone tunnel enlargement was not observed at radiological examination. However, in the future, long term clinical and radiological studies as well as a biochemical study will be required.

There have been several reports on reconstructing techniques using BTB and hamstring for the ruptured ACL [17,18]. However, these procedures did not represent an anatomical reconstruction in a tunnel position or routes strictly. Moreover, 10 mm BTB graft is bigger than one third of patella tendon width of Asian patient. To solve these problems, an anatomical double bundle ACL reconstructive procedure using BTB and gracilis composite autograft was modified and improved.

## Conclusion

This modified technique uses a BTB and gracilis composite autograft for anatomical double bundle ACL reconstruction. The present procedure can have advantages of using BTB graft for double bundle technique, and reduce the risk of tunnel enlargement. It would be a useful treatment method for those ACL deficient patients who have small or medium width of patella tendon such as Asian people or females. This technique is also applicable to revision surgery.

## Abbreviations

ACL: Anterior cruciate ligament; BTB: Bone-patellar tendon-bone; AMB: Anteromedial bundle; PLB: Posteromedial bundle.

## Competing interests

The authors declare that they have no competing interests.

## Authors' contributions

TT participated in the design of the study and performed surgery. KY and NY carried out surgery. EC participated in the design and coordination. All authors read and approved the final manuscript.

## Authors' information

M.D, Ph.D

Assistant professor of Division of Orthopedic Surgery, Department of Medicine of Sensory and Motor Organs, Faculty of Medicine, University of Miyazaki

Member of Japanese Orthopaedics Society

Member of Japanese Orthopaedics Society for Sports Medicine

Member of Japanese Society of Clinical Sports Medicine

Member of Japanese Orthopaedics Society Knee, Arthroscopy and Sports Medicine
